# Influenza NA and PB1 Gene Segments Interact during the Formation of Viral Progeny: Localization of the Binding Region within the PB1 Gene

**DOI:** 10.3390/v8080238

**Published:** 2016-08-20

**Authors:** Brad Gilbertson, Tian Zheng, Marie Gerber, Anne Printz-Schweigert, Chi Ong, Roland Marquet, Catherine Isel, Steven Rockman, Lorena Brown

**Affiliations:** 1 Department of Microbiology and Immunology, The University of Melbourne at the Peter Doherty Institute of Infection and Immunity, Parkville 3010, Victoria, Australia; bg@unimelb.edu.au (B.G.); tianchi.zheng@gmail.com (T.Z.); Steve.Rockman@seqirus.com (S.R.); 2 Architecture et Réactivité de l’ARN, Université de Strasbourg, CNRS, IBMC, 15 rue René Descartes, Strasbourg 67084, France; m.gerber@ibmc-cnrs.unistra.fr (M.G.); A.schweigert@ibmc-cnrs.unistra.fr (A.P.-S.); r.marquet@ibmc-cnrs.unistra.fr (R.M.); c.isel@ibmc-cnrs.unistra.fr (C.I.); 3 Seqirus, 63 Poplar Rd, Parkville 3052, Victoria, Australia; Chi.Ong@seqirus.com; 4 Unité de Génétique Moléculaire des Virus à ARN, Département de virologie, Institut Pasteur, Paris 75005, France

**Keywords:** influenza virus, packaging, reassortment, RNA-RNA interaction, gene segments, viral polymerase, competitive transfection

## Abstract

The influenza A virus genome comprises eight negative-sense viral RNAs (vRNAs) that form individual ribonucleoprotein (RNP) complexes. In order to incorporate a complete set of each of these vRNAs, the virus uses a selective packaging mechanism that facilitates co-packaging of specific gene segments but whose molecular basis is still not fully understood. Recently, we used a competitive transfection model where plasmids encoding the A/Puerto Rico/8/34 (PR8) and A/Udorn/307/72 (Udorn) PB1 gene segments were competed to show that the Udorn PB1 gene segment is preferentially co-packaged into progeny virions with the Udorn NA gene segment. Here we created chimeric PB1 genes combining both Udorn and PR8 PB1 sequences to further define the location within the Udorn PB1 gene that drives co-segregation of these genes and show that nucleotides 1776–2070 of the PB1 gene are crucial for preferential selection. In vitro assays examining specific interactions between Udorn NA vRNA and purified vRNAs transcribed from chimeric PB1 genes also supported the importance of this region in the PB1-NA interaction. Hence, this work identifies an association between viral genes that are co-selected during packaging. It also reveals a region potentially important in the RNP-RNP interactions within the supramolecular complex that is predicted to form prior to budding to allow one of each segment to be packaged in the viral progeny. Our study lays the foundation to understand the co-selection of specific genes, which may be critical to the emergence of new viruses with pandemic potential.

## 1. Introduction

The influenza A virus (IAV) genome comprises eight individual ribonucleoprotein (RNP) complexes each with a single-stranded negative-sense viral RNA (vRNA) segment encoding one or more proteins. In each RNP complex, the vRNA is encapsidated by nucleoprotein (NP) and the 3′ and 5′ termini show inverted complementarity and associate to form a double stranded structure referred to as the panhandle [1]. On each segment, the termini are attached to the viral RNA-dependent RNA polymerase complex consisting of three subunits: polymerase basic protein 1 (PB1), polymerase basic protein 2 (PB2) and polymerase acidic protein (PA) [2,3]. Each RNP is thus an independent unit for transcription and replication of the associated vRNA [4,5].

Each gene segment encodes at least one essential viral protein, therefore complicating packaging of the viral genome, as the virus must incorporate one copy of each vRNA to be fully infectious. Electron microscopy [6] and tomography [7,8,9] have repeatedly revealed that the eight RNPs of budding IAVs are organized in a specific ‘7 + 1’ pattern, with seven RNPs surrounding a central segment. Recently, single-molecule fluorescent in situ hybridization analysis directly showed that only one copy of each RNA segment is packaged into most viral particles [10]. Similarly, in a reverse genetics system that included two virus-like RNAs encoding different reporter proteins within identical vRNA segments, plaques formed from progeny virions displayed only one or the other reporter protein but not both, further showing that individual influenza virions incorporate single, not multiple, copies of each vRNA segment [11]. The mechanism underlying a now well established selective rather than random packaging process has recently started to be unveiled. *Cis*-acting packaging sequences, located in both the 3′ and 5′ noncoding regions as well as the terminal coding regions of each RNA segment have been identified in all eight vRNA segments, for the A/WSN/33 (H1N1) and A/Puerto Rico 8/1934 (H1N1) (PR8) strains (for reviews, see [12,13]). Mutations introduced into these sequences dramatically reduce the incorporation efficiency of both the mutated vRNA and of other vRNAs into progeny virions, highlighting the importance of these regions and suggesting the existence of intersegment interactions critical for proper packaging [14,15,16]. Direct vRNA/vRNA interactions as guides for selective genome packaging [12] warranted investigation. Using an in vitro RNA/RNA interaction assay, the eight vRNA segments from the human H3N2 (A/Moscow/10/99) and avian H5N2 (A/Finch/England/205/91) viruses were shown to be organized into a limited number of strain-specific supramolecular assemblies maintained by a network of intermolecular interactions where each vRNA segment interacted with at least one other partner [17,18]. In the case of H5N2, sequences involved in vRNA/vRNA interactions were found to be non-conserved and not exclusively located at the termini but rather spread along the viral RNPs (vRNPs) [18].

While the segmented nature of the viral genome requires a highly sophisticated packaging strategy, it provides IAVs with a significant evolutionary advantage. Indeed, it allows genetic reassortment when two or more influenza viruses co-infect the same cell, creating hybrid viruses containing a mixture of segments from the two parental strains. This process is a major contributing factor in the emergence of novel pandemic strains, whereby IAVs acquire novel hemagglutinin (HA) and neuraminidase (NA) to which the human population is essentially naïve. Although the theoretical number of genotypes that can emerge from natural or experimental reassortment is 256 (2^8^), the whole panel of different gene combinations has never been observed [19,20,21] and certain genes tend to co-segregate [20,22,23,24], suggesting that genetic reassortment is biased [13,22,24]. Documented incompatibilities between viral proteins or viral and host proteins explain the absence of some gene constellations [21,25,26,27,28] and viruses engineered to differ slightly in gene but not protein sequences have been found to reassort very efficiently and without genetic bias [29]. However, it is possible that incompatibilities at the genomic level, most likely between packaging signals, may also shape the final constellation of genes. The ability to rescue efficiently replicating viruses by reverse genetics that do not emerge during reassortment would support this. Hence, we have postulated that the drivers of packaging of influenza gene segments can also impact on the reassortment process. Accordingly, Essere et al. [22] recently demonstrated that during in vitro reassortment between a human H3N2 and an avian H5N2 virus, the HA and M gene segments co-segregate and that genetic reassortment is limited by sub-optimal compatibilities between packaging signals. Similarly, it was observed that H3N2 vaccine seeds, derived by classical reassortment with the egg-adapted PR8 H1N1 virus and selected with antisera to PR8 to yield progeny containing H3N2 HA and NA, frequently contain the PB1 gene from the H3N2 strain [30,31,32]. This finding was recapitulated using the model seasonal H3N2 virus Udorn and is noteworthy because, despite its preferential selection in the reassortment progeny, reverse genetics experiments showed that the virus containing the Udorn PB1, in addition to Udorn HA and NA, did not replicate as well as the corresponding virus containing the PR8 PB1 segment [32]. This indicated that inclusion of the Udorn PB1 gene was not driven by selection of the fittest virus.

To study this phenomenon a competitive nine-plasmid transfection model was developed, where plasmids encoding Udorn HA and NA and the other six genes of PR8 were provided together with an additional Udorn PB1. When given the choice of PB1 genes, packaged progeny viruses predominantly contained the Udorn PB1. Similar experiments linked the preferential co-segregation of Udorn PB1 to the Udorn NA and not HA segment [24]. Therefore it was postulated that incorporation of H3N2 PB1 into vaccine seeds was likely due to this same co-selection mechanism with potential pairing with the H3N2 NA segment. Additional competitive transfection experiments using chimeric PB1 genes in which the 3′ and 5′ packaging sequences were swapped between PR8 and Udorn PB1 genes revealed that incorporation of PR8 PB1 was governed by previously described packaging sequences present at the 3′ end of the vRNA. However, the co-selection of Udorn NA and PB1 segments was not directed by these terminal packaging sequences but rather through interactions involving the internal coding region of the PB1 gene [24].

In this study, we investigate interactions between the PB1 and NA genes that potentially explain why H3N2 NA and PB1 co-segregate. We have created additional chimeric PB1 genes to further delineate the regions within the central coding area of the Udorn PB1 gene that interacts with the Udorn NA gene. This work has identified a region of the PB1 gene that influences the association between viral genes to direct their co-selection during reassortment. This region potentially encompasses sequences driving key RNP-RNP interactions within the supramolecular complex that is predicted to form prior to budding to allow one of each segment to be packaged in the viral progeny.

## 2. Materials and Methods

### 2.1. Cells and Media

Human embryonic kidney (293T) cells and Madin-Darby canine kidney (MDCK) cells were sourced from an existing collection in the Department of Microbiology and Immunology, The University of Melbourne, Australia. 293T cells were grown in Dulbecco’s modified Eagle’s medium (DMEM) (Life Technologies, Mulgrave, Victoria, Australia) and MDCK cells were grown in RPMI-1640 (Sigma Aldrich, Castle Hill, NSW, Australia). Both media were supplemented with 10% heat-inactivated fetal calf serum (FCS, Life Technologies), 2 mM l-glutamine (Sigma Aldrich), 2 mM sodium pyruvate (Thermo Fisher, Scoresby, Victoria, Australia), 24 μg/mL gentamicin (Pfizer, West Ryde, NSW, Australia), 50 μg/mL streptomycin (Life Technologies) and 50 IU/mL penicillin (Life Technologies). Co-cultures of 293T cells and MDCK cells for transfection were established in Opti-MEM (Life Technologies) with 50 μg/mL streptomycin and 50 IU/mL penicillin.

### 2.2. Construction of Reverse-Engineered Influenza Viruses

Individual gene segments from PR8 and Udorn viruses were reverse transcribed and cloned into pHW2000 plasmids (St. Jude Children’s Hospital, Memphis, TN, USA). Viruses were reverse engineered using the eight-plasmid system developed by Hoffmann et al. [33]. The reverse genetics-derived viruses consisted of a parental or chimeric PB1 gene in a genetic background comprising six gene segments from PR8 virus (PB2, PA, HA, NP, M, NS) and the NA gene segment derived from Udorn virus. This backbone virus is referred to as PR8(Ud-NA) and those expressing chimeric PB1 segments collectively as PR8(Ud-NA, chimeric PB1) or specifically by the chimeric PB1 name e.g., PPU or PPpup. The origin of each third of the PB1 gene is denoted by P (PR8) or U (Udorn). Uppercase nomenclature has been used to indicate larger thirds of the PB1; lowercase nomenclature used to indicate the smaller three sections of the last third of the PB1. Rescued viruses were amplified from transfection supernatants in 10-day embryonated hen’s eggs. Infectious allantoic fluid was titrated for virus content by plaque formation in MDCK cells [34] and stored at −80 °C.

### 2.3. Construction of Plasmids Expressing Chimeric PB1 Gene Segments

Briefly, plasmids expressing chimeric PB1 genes were created by the construction of fragments of PR8 and Udorn PB1 genes representing approximate thirds of the respective gene and had a 20 nucleotide overlap with the adjacent sequence, conserved in both virus strains. These were amplified by PCR using section-specific primers and joined together by another round of PCR using segment-specific primers containing AarI restriction sites [35]. Full-length chimeric products were then cloned into the pHW2000 expression vector. All clones were confirmed by full-length sequencing. The resulting chimeric PB1 genes were designated PPU, PUU, PUP, UUP, UPP and UPU (representing the PR8 or Udorn origin of each third of the gene). This same approach was used to construct additional chimeras in which the 5′ third was further subdivided into approximate thirds to yield PPppu, PPpuu, PPpup, PPuup, PPupp and PPupu (where the lowercase letters designate the origin of thirds within the last 5′ section). The primer sequences are available upon request.

### 2.4. Minigenome Assay for Polymerase Activity

A β-lactamase (BLA) reporter assay [36] was used to compare the intrinsic activities of viral RNP complexes as previously described [32]. Briefly, the pCAGGS-BLA reporter plasmid (2 ng/well), in which the BLA gene is flanked by influenza virus segment untranslated regions (UTRs; derived from H1 HA), was transfected into 293T cells, together with 2 ng of four pHW2000 plasmids expressing either the NP gene or one of the three influenza virus polymerase genes (PB1, PB2, or PA); the PB1 being either parental or chimeric. Transfected cells were incubated at 37 °C in 5% CO_2_. At 24 h post transfection, the β-lactamase produced was detected, after lysis of cells, by the addition of the LyticBLazer-FRET B/G substrate (Life Technologies) according to the manufacturer’s instructions. Fluorescence was read using a ClarioStar microplate reader (BMG Labtech, Mornington, Victoria, Australia). The relative polymerase activity was calculated as follows: (530 nm/460 nm ratio of the sample)/(530 nm/460 nm ratio of untransfected control).

### 2.5. Determination of Viral Replication Kinetics

The replication characteristics of the reverse-engineered viruses were determined by infecting MDCK cells at a multiplicity of infection (MOI) of 0.01 plaque forming units (pfu)/cell. Following 1 h absorption (at *t* = 0 h) the inoculum was removed and cells were washed and incubated at 37 °C, 5% CO_2_ in RPMI-1640 (Sigma Aldrich) supplemented with 2 mM l-glutamine, 2 mM sodium pyruvate, 24 μg/mL gentamicin, 50 μg/mL streptomycin and 50 IU/mL penicillin and 1 μg/mL of TPCK-trypsin (Worthington Biochemical Corporation, Lakewood, NJ, USA). Cell culture supernatants were harvested at various time points post infection and stored at −80 °C for analysis. Viral titres of the supernatants were determined by plaque formation on confluent monolayers of MDCK cells.

### 2.6. Nine-Plasmid Competitive Transfections

These were undertaken using a modified version of the eight-plasmid reverse genetics system [33] as described previously [24]. Plasmids encoding the six internal PR8 genes and the Udorn NA gene were provided with additional competing PB1 genes. One μg of each plasmid was mixed with FuGENE6 transfection reagent (Promega, Alexandria, NSW, Australia) in Opti-MEM and added to a co-culture of 239T and MDCK cells. Six hours post-transfection, media was replaced with Opti-MEM supplemented with 50 μg/mL streptomycin and 50 IU/mL penicillin. Twenty-four hours later, TPCK-trypsin (1 μg/mL) was added and the supernatant harvested after a further 42 h and stored at −80 °C. To determine the incorporation frequencies of the PB1 gene in progeny viruses, the transfection supernatant was plaqued in MDCK cells. Randomly chosen plaques were picked by sampling through the agarose and resuspended in 0.05% Triton-X (Sigma Aldrich). The source of the competing gene segments was identified by gene-specific reverse transcription PCR (RT-PCR). The final data are derived from at least three independent experiments.

### 2.7. Gene-Specific RT-PCR

SensiFast probe no-ROX one-step RT-PCR Kit (Bioline, Alexandria, NSW, Australia) was used for gene identification. Each 20 μL reaction was performed using 5 μL of plaque-picked virus suspension, 10 μL of 2× SensiFast No-ROX one-step mix, 0.2 μL of reverse transcriptase, 0.4 μL of Ribosafe RNase inhibitor, 0.8 μL of each 10 μM gene-specific forward and reverse primer and 0.08 μL of each 25 μM gene-specific probe. The reverse transcription reaction was incubated for 10 min at 45 °C. Amplification and detection was performed using a Bio-Rad CFX96 RT-PCR system (Gladesville, NSW, Australia). Reaction conditions, primers (Geneworks, Thebarton, SA, Australia) and TaqMan (Integrated DNA Technologies, Baulkham Hills, NSW, Australia) probe sequences are available on request.

### 2.8. In Vitro Transcription and Electrophoretic Mobility Assay

DNA corresponding to the chimeric PB1 or Udorn NA genes was inserted between the BsmBI (ThermoFisher Scientific, Illkirch, France) sites of a pUC2000 vector after excision from the corresponding pHW2000 plasmid. These vectors were obtained as previously described and contain a T7 promoter, the cloning cassette of pHW2000 containing two BsmBI sites and unique Bsh1236I or Ecl36II restriction sites between the PstI and EcoRI sites [9]. In vitro transcription of PB1 and NA vRNAs was performed with 30 µg of Bsh1236I (ThermoFisher Scientific) or Ecl36II (New England BioLabs, Evry, France) linearized plasmid, respectively, in a 300 µL reaction volume containing 40 mM Tris-HCl pH 7.5, 50 mM NaCl, 15 mM MgCl_2_, 1.7 mM spermidine (Sigma, Saint-Quentin Fallavier France), 5 mM 1,4-dithiothreitol (DTT) (USB, Santa Clara, CA, USA), 4 mM of each nucleotide triphosphate (NTP) (Sigma), 400 U RNasin (Promega), 0.01% Triton X-100 (Sigma) and 3 µL of T7 RNA polymerase (made in-house). After 3 h of incubation, samples were treated with 50 U of RNase-free DNase I ((Roche, Meylan, France)) for 2 h at 37 °C, vRNAs were extracted with phenol/chloroform (Carl Roth GmbH, Karsruhe, Germany), ethanol precipitated and purified on a TSK G2000SW column (Tosoh Bioscience GmbH, Stuttgart, Germany). The integrity of the vRNAs was checked by denaturing polyacrylamide gel electrophoresis. Pairs of purified vRNAs (2 pmol each) were denatured for 2 min at 90 °C in 8 µL of water and cooled on ice. Two microliters of 5-fold concentrated buffer (final concentration: 50 mM sodium cacodylate (Sigma), pH 7.5; 40 mM KCl and 0.1 mM MgCl_2_) were added, the samples were incubated for 30 min at 55 °C and then analysed on 0.8% agarose gels containing 0.01% (w/v) ethidium bromide (Carl Roth GmbH) after addition of 5 µL of loading buffer [40% (v/v) glycerol (Sigma); 0.05% (w/v) xylene cyanol (USB, Santa Clara, CA, USA); 0.05% bromophenol blue (Sigma)]. Native gel electrophoresis was performed for 4 h at 150 V at 4 °C in a buffer containing 50 mM Tris, 44.5 mM borate and 0.1 mM MgCl_2_. Gels were analyzed using a Gel Doc (Bio-Rad) imager and the ImageLab (Bio-Rad) software. The RNA weight fraction (%) of each band in a lane was determined and the percentage of intermolecular complex was calculated by dividing the weight fraction of the corresponding band by the sum of the weight fractions of all bands in the lane.

## 3. Results

### 3.1. Viruses Expressing Chimeric PB1 Genes Show No Major Differences in Replication in Vitro

**Figure 1:**
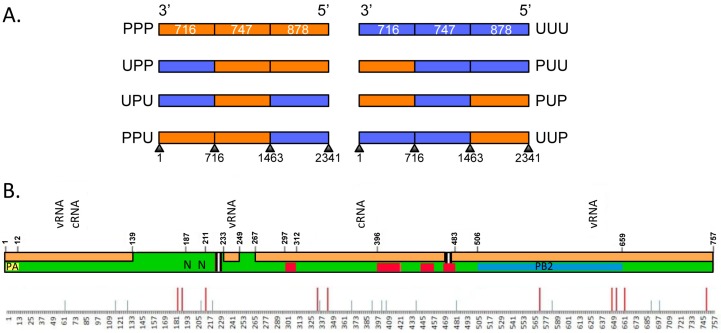
Structure of chimeric PB1 plasmids and functional domains of the PB1 gene. (**A**) Schematic representation of chimeric PB1 plasmid constructs. The origin of each third of the gene is colour-coded, orange represents PR8 (P) and blue represents Udorn (U) sequences. The nucleotide length and boundaries of each section are indicated. Influenza vRNA are numbered from 3′ to 5′ according to international nomenclature; (**B**) Schematic representation of the functional domains of the PB1 protein highlighting the amino acid differences between Udorn and PR8 PB1. Polymerase acidic protein (PA) binding domain (yellow); Nuclear localization signals (N); conserved RNA-dependent RNA polymerase motifs (red); Polymerase basic protein 2 (PB2) binding domain (blue); interaction with viral RNA (vRNA) and complementary RNA (cRNA) (orange). Amino acid differences between PR8 and Udorn PB1 proteins are shown below the schematic with smaller grey lines indicating the location of conservative amino acid changes and bigger red lines indicating non-conservative amino acid changes.

To investigate the interaction between the Udorn NA gene and the central coding region of the Udorn PB1 gene, we used overlapping PCR to divide the PB1 gene segment into approximate thirds and create plasmids encoding a series of chimeric PB1 genes containing sections swapped between Udorn PB1 and PR8 PB1. The six possible chimeric PB1s produced by this process are illustrated in Figure 1A and are denoted as UPP, UPU, UUP, PPU, PUP or PUU depending on the PR8 (P) or Udorn (U) origin of each third of the gene. In addition, plasmids encoding entirely PR8 (PPP) and Udorn (UUU) PB1 genes were included as controls. Figure 1B shows a representative schematic of the PB1 protein encoding 757 amino acids, highlighting various functional domains. The first section contains the PA binding domain [37] and two discrete nuclear localisation signals [38]. The middle section contains four regions of high conservation in all RNA-dependent RNA polymerases [39,40], and the last section contains the PB2 binding domain [37]. Interactions of the PB1 protein with vRNA and complementary RNA (cRNA) span all three sections [41]. Efforts to minimise any potential disruption of the functional domains were made by placing the boundary sites in regions of homology between PR8 and Udorn PB1s. Table 1 summarises the location, length, nucleotide homology and amino acid differences between PR8 and Udorn in respect to the three sections of the chimeric PB1 genes. There are only 25 differences out of 757 amino acids across the entire PR8 and Udorn PB1 proteins despite a relatively low nucleotide homology of 84%.

**Figure 2:**
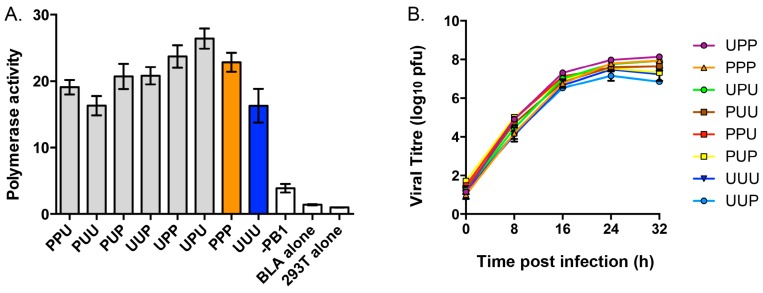
Effect of chimeric PB1 genes on polymerase activity and replication of PR8(Ud-NA, chimeric PB1) viruses. (**A**) Minigenome assays were performed in 293T cells transfected with the pCAGGS-BLA reporter gene, plasmids expressing the PR8 PB2, PA and NP genes, together with the indicated chimeric PB1 gene or wild-type PR8 (PPP; orange) or Udorn (UUU; blue) PB1 genes. The polymerase activity of each ribonucleoprotein (RNP) complex was measured after 90 min and compared to a transfection mixture without a PB1 gene (-PB1), cells transfected with the BLA reporter alone (BLA alone) and untransfected cells (293T alone). The data represents the mean and standard error of two independent experiments; (**B**) MDCK cells were infected with viruses containing chimeric PB1 genes at a multiplicity of infection (MOI) of 0.01. At the indicated time points, the amount of infectious virus released into the supernatant was determined by plaque assay. The data represent the mean and standard error of three individual experiments.

To establish whether the chimeric PB1s were functional, a BLA reporter assay was used in cells cotransfected with plasmids expressing parental or chimeric PB1 in addition to those expressing each of the RNP components (PA, PB2, NP) of PR8 virus. Measurement of the cleavage of substrate by the expressed BLA enzyme (Figure 2A), which correlates with polymerase activity, revealed that an entirely PR8 RNP has a higher polymerase activity than one containing the PB1 from Udorn virus as expected [32]. Notably, all of the chimeric PB1s formed RNP complexes that were functional and were not significantly different from either PR8 or Udorn PB1-containing RNPs.

Eight-plasmid reverse genetics was then used to construct viruses expressing chimeric PB1 genes on the backbone of PR8(Ud-NA) virus, which contains the NA gene from Udorn virus and all other genes from PR8 virus. This backbone was chosen for all future competitive transfection experiments as it was postulated that the Udorn NA RNP would allow co-selection of specific chimeric PB1 genes that contained the putative PB1-NA nucleotide binding motif. All of the six PR8(Ud-NA) viruses expressing chimeric PB1 genes could be rescued. When the viral growth kinetics of these viruses were assessed in MDCK cells (Figure 2B), no statistically significant differences in the replicative efficiency was observed over the first 16 h between PR8(Ud-NA) virus containing a PR8 PB1 (PPP), its PR8(Ud-NA, PB1) counterpart containing a Udorn PB1 (UUU), or any of the PR8(Ud-NA) viruses expressing chimeric PB1 genes. This meant that the PR8(Ud-NA) backbone would allow future PB1 competitive transfection experiments to be investigated in the absence of differences in the intrinsic replicative capacity of the competing progeny. This formed a key part of the rationale for using the PR8(Ud-NA) backbone as we had previously shown with viruses additionally expressing the Udorn HA that PR8(Ud-HA,NA,PB1) displays vastly inferior replicative efficiency compared to PR8(Ud-HA,NA) [32], making the PR8(Ud-HA,NA) backbone unsuitable to determine outcomes due to gene co-segregation rather than replicative advantage. At the later time points of 24–32 h only one virus, which expressed UUP, showed statistically lower replication than the other viruses (lower compared to UPP, PPP and UPU at 24 h, and compared to all except UUU at 32 h) and it is possible that this chimeric PB1 may not be represented as well in the progeny of competitive transfections due to this slight decrease in replicative capacity.

### 3.2. Nine Plasmid Competitive Transfections Reveal Chimeras Expressing Any Section of Udorn PB1 Are Preferentially Selected Over PR8 PB1 in Progeny Virus

**Figure 3:**
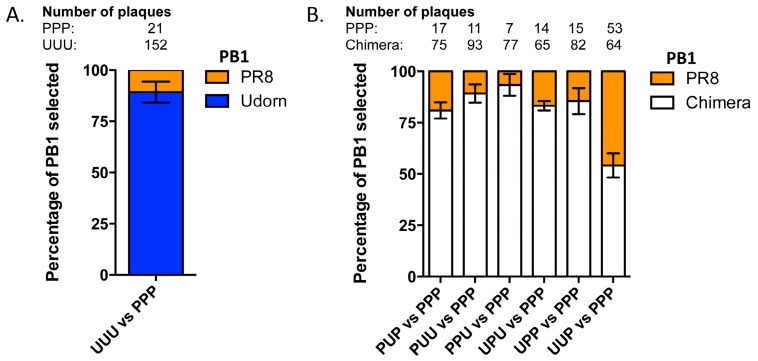
Frequency of competing genes incorporated in progeny viruses resulting from nine-plasmid competitive transfection assays. Nine-plasmid competitive transfections were performed on a PR8(Ud-NA) background competing (**A**) wild-type PR8 (orange) and Udorn (blue) PB1 genes or (**B**) the wild-type PR8 PB1 gene (PPP; orange) against chimeric PB1 genes (white) as indicated. The origin of the PB1 gene segment from a random selection of progeny plaques was determined by gene-specific RT-PCR. Total numbers of specific plaques determined from each competition are indicated. The data represent the mean and standard error of 3–5 individual experiments.

To attempt to define the region of the PB1 driving co-selection of this gene with the Udorn NA we used a nine-plasmid transfection system in which plasmids encoding the genetic backbone of PR8(Ud-NA) were provided together with plasmids encoding different PB1 genes. The resulting viral progeny in the transfection supernatants were then analysed by RT-PCR from plaque-picks to determine the source of the competing PB1 gene. Initially we competed the wildtype PR8 and Udorn PB1 genes to re-affirm data we have published previously [24] showing that the Udorn PB1 gene is incorporated at a significantly higher frequency than PR8 PB1 (*p* < 0.0001) in the presence of the Udorn NA gene on this background (Figure 3A).

The chimeric PB1 genes were then competed with the PR8 PB1 gene in the same system. At least three independent experiments were performed for each competitive transfection and the frequencies of the PB1 genes selected in each case were averaged across the different experiments (Figure 3B). Chimeric PB1s with a Udorn section in any position were selected at a greater frequency than that of the PR8 PB1 (PUP; *p* = 0.0004, PUU; *p* = 0.0002, PPU; *p* = 0.0003, UPU; *p* < 0.0001, UPP; *p* = 0.0014 (Student’s *t*-test)). This was in agreement with selection of Udorn PB1 over PR8 PB1 in PR8(Ud-NA) backbone virus. The exception to this was the chimeric UUP PB1 which was selected at a similar frequency (54% ± 5%; *p* = 0.3577 (Student’s *t*-test)) to PPP PB1. This was not surprising as PR8(Ud-NA, UUP PB1) demonstrated a significantly inferior intrinsic replication efficiency compared to PR8(Ud-NA) expressing PPP PB1 in MDCK cells (Figure 2B). As a result, any selection due to the presence of a Udorn section in UUP PB1 could potentially be offset by the replicative advantage of PR8(Ud-NA) in the transfection culture. The chimeric PPU PB1 containing the last section of Udorn had the highest preferential incorporation frequency over PPP PB1 (93% ± 4%).

### 3.3. The Last Third of PB1 RNA Is Driving Preferential Co-Selection of Udorn PB1 and Udorn NA

**Figure 4:**
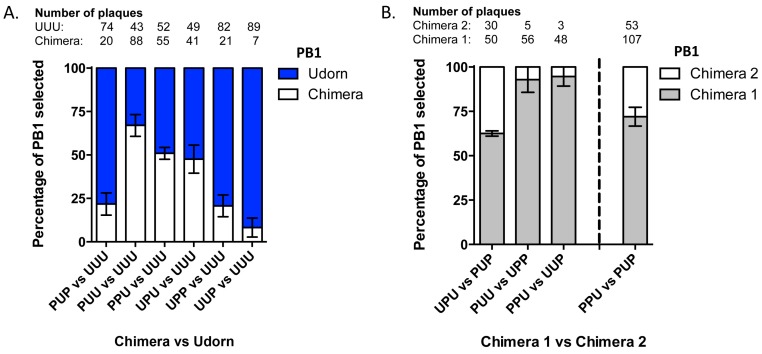
Frequency of competing genes incorporated in progeny viruses resulting from nine-plasmid competitive transfection assays. Nine-plasmid competitive transfections were performed on a PR8(Ud-NA) background competing (**A**) the wild-type Udorn PB1 gene (UUU; blue) against chimeric PB1 genes (white) as indicated or (**B**) two chimeric PB1 genes as indicated. The origin of the PB1 gene segment from a random selection of progeny plaques was determined by gene-specific RT-PCR. Total numbers of specific plaques determined from each competition are indicated. The data represent the mean and standard error of 2–5 individual experiments.

Chimeric PB1 genes were then competed against the Udorn PB1 gene in the nine-plasmid transfection system. Again, three or more replicate experiments were performed for each competition and the frequency of each chimera averaged (Figure 4A). Unlike the competitions against PR8 PB1, chimeras displayed hierarchical selection against Udorn PB1. Three of the chimeras displayed comparable or even greater preferential selection to UUU PB1. Of these, the chimera selected at the highest frequency was PUU (67% ± 6%; *p* = 0.0084), containing the middle and last sections of Udorn, followed by PPU (51% ± 3%; *p* = 0.7137) and UPU (48% ± 6%; *p* = 0.6964 (Student’s *t*-test)). PUP, UPP and UUP chimeras were all selected at a significantly lower frequency to the wild-type Udorn PB1 (<25%; PUP; *p* = 0.0033, UPP *p* = 0.0027, UUP; *p* = 0.0004 (Student’s *t*-test)). The chimeric genes that showed equivalent selection to UUU had the last third from Udorn PB1 in common, suggesting this region may be driving the co-selection.

To confirm the importance of the last third of Udorn PB1 in cosegregation with Udorn NA, the different chimeras were then competed head-to-head in nine-plasmid transfections. The initial approach was to compete sets of chimeras that contained ‘complementary’ regions of the PB1. For example, competing UPU against PUP, PUU against UPP and PPU against UUP. In these experiments the PB1 having Udorn first and last sections (UPU) was preferentially selected over the middle (PUP; *p* = 0.0066); Udorn middle and last sections (PUU) was preferentially selected over the first (UPP; *p* = 0.0136); and the Udorn last section (PPU) was preferentially selected over the first and middle (UUP; *p* = 0.0071 (Student’s *t*-test)) (Figure 4B). Once again the chimeras that were preferentially selected in these competitions all contained the last Udorn PB1 section, although the UPU chimera was not selected as strongly when competed against PUP (63% ± 1%) as was PUU (93% ± 5%) and PPU (95% ± 3%) when competed against their complementary PB1s. This indicates a possible involvement of the middle section or conversely, a negative effect on selection of the first section when paired with the last section (UPU). To confirm whether the middle or last section was more important in driving co-selection with the Udorn NA it was decided to compete the PUP and PPU chimeras head-to-head. Five independent transfections competing these specific chimeras were performed and in all competitions the PPU chimera, containing the last section of the Udorn PB1, was preferentially selected (*p* = 0.0011 (Student’s *t*-test)) (Figure 4B), indicating the region between nucleotides 1464–2341 of PB1, corresponding to the 5′ end of the vRNA, is the likely driver of co-segregation with Udorn NA and may contain a region by which the two RNPs interact.

### 3.4. Viruses Expressing 5′ Chimeric PB1 Genes Show No Major Differences in Replication in Vitro

**Figure 5:**
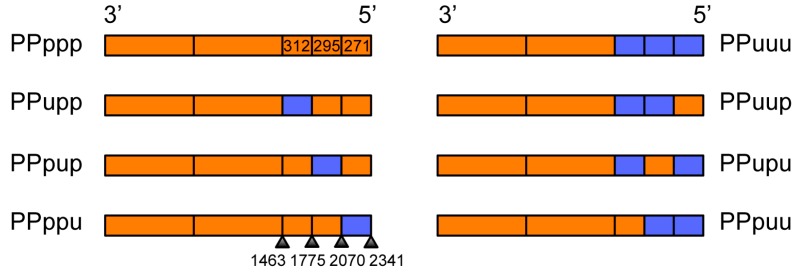
Structure of 5′ chimeric PB1 plasmids. Schematic representation of 5′ chimeric PB1 plasmid constructs showing the origin of each section of the gene; orange represents PR8 (P) and blue represents Udorn (U) sequences. In the nomenclature for the chimeras, uppercase indicates the origin of the larger thirds of the PB1 (all PR8); lowercase indicates the origin of the smaller three sections of the last third of the PB1. The nucleotide length and boundaries of each 5′ section is indicated.

As the last section of the PB1 was crucial for the PB1-NA gene co-segregation we wished to further dissect this region by creating chimeras of the last 5′ section. Once again the strategy was to divide this section into approximate thirds using overlapping PCR and create a panel of 5′ chimeric PB1 plasmids that we could compete in the nine-plasmid transfection system. A schematic of the 5′ chimeric PB1 plasmids is shown in Figure 5. These 5′ chimeras contained the first and middle thirds of PR8 (P), with the last third divided into three sections and swapped interchangeably between PR8 (p) and Udorn (u), designated PPupp, PPpup, PPppu etc. Uppercase nomenclature has been used to indicate larger thirds of the PB1; lowercase nomenclature used to indicate the smaller three sections of the last third of the PB1.

Table 2 summarises the location, length, nucleotide homology and amino acid differences between PR8 and Udorn in respect to the three sections within the 5′ third of the chimeric PB1 genes. Eight of the 25 amino acid differences across the entire PR8 and Udorn PB1 proteins occur in the last third of the gene. At the nucleotide level the last third of the PB1 gene showed 83.7% homology between Udorn and PR8. Within this third, the middle section displayed the lowest homology between PR8 and Udorn viruses.

**Figure 6:**
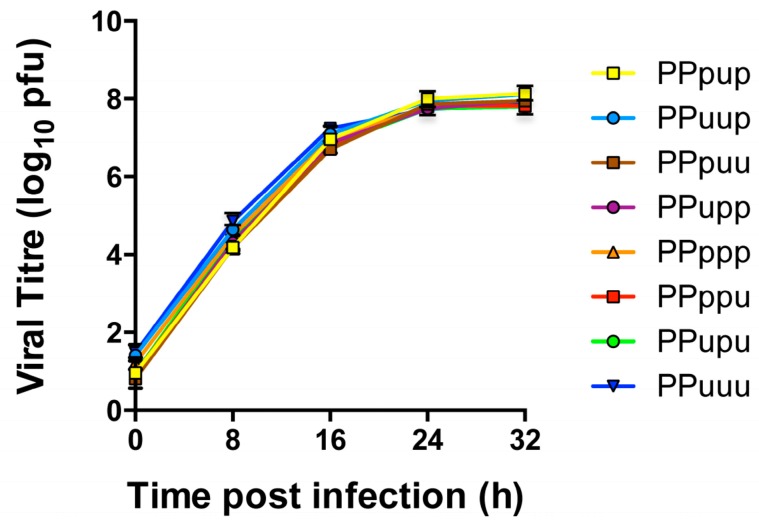
Growth kinetics of reverse genetics-derived PR8(Ud-NA, 5′ chimeric PB1) viruses. Madin-Darby canine kidney (MDCK) cells were infected with viruses containing 5′ chimeric PB1 genes at an MOI of 0.01. At the indicated time points, the amount of infectious virus released into the supernatant was determined by plaque assay. The data represent the mean and range of two individual experiments.

Eight-plasmid reverse genetics was again used to engineer these 5′ chimeric PB1 genes onto the backbone of PR8(Ud-NA). All of the PR8(Ud-NA) viruses expressing 5′ chimeric PB1 genes were successfully rescued. The viral growth kinetics of these viruses were again assessed in MDCK cells. Viral loads determined from samples of culture supernatants taken at 8 h intervals showed there were no significant differences in the viral replication of the respective 5′ chimeric viruses (*p* = 0.1919 (two-way analysis of variance (ANOVA) repeated measures)) (Figure 6). As a result selection of chimeras containing specific Udorn sections in subsequent competitive transfections could be attributed solely to co-selection of the Udorn region with the Udorn NA without any confounding effects of intrinsic replicative advantage.

### 3.5. The PB1 RNA Segment Containing Nucleotides 1776–2070 Is Driving Preferential Coselection of Udorn PB1 and Udorn NA

**Figure 7:**
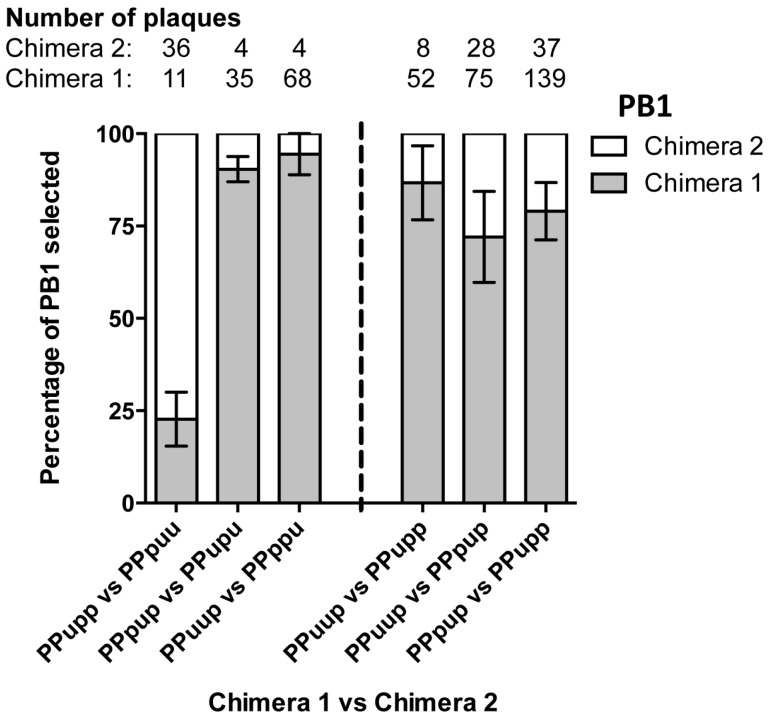
Frequency of competing chimeric genes incorporated in progeny viruses resulting from nine-plasmid competitive transfection assays. Nine-plasmid competitive transfections were performed on a PR8(Ud-NA) background competing two 5′ chimeric PB1 genes as indicated. The origin of the PB1 gene segment from a random selection of progeny plaques was determined by gene-specific RT-PCR. Total numbers of specific plaques determined from each competition are indicated. The data represent the mean and standard errors of 2–5 individual experiments.

Sets of complementary 5′ chimeric PB1 genes on a PR8(Ud-NA) backbone were then competed head-to-head in nine-plasmid transfections. Initially we competed PPupp against PPpuu, PPpup against PPupu and PPuup against PPppu. In these experiments, the Udorn middle and last 5′ sections (PPpuu) were preferentially selected over the first 5′ section (PPupp; *p* = 0.0339); the Udorn middle 5′ section (PPpup) was preferentially selected over the first and last 5′ sections (PPupu; *p* = 0.0035); and the Udorn first and middle 5′ sections (PPuup) were preferentially selected over the last 5′ section (PPppu; *p* = 0.0077 (Student’s *t*-test)) (Figure 7 left panel).

Chimeras that were preferentially selected in these competitions all contained the middle section of the last third of the Udorn PB1 gene. To confirm this observation and to investigate whether the first 5′ section had any co-involvement in selection, a set of additional competitions were performed (Figure 7, right panel), firstly keeping the first 5′ section constant and testing the requirement for the middle section (PPuup versus PPupp). In these competitions the chimera that also contained the middle 5′ Udorn section was preferentially selected (87% ± 6%, *p* = 0.0352) confirming the importance of this component of the gene. Competitions to investigate any co-involvement of the first 5′ Udorn region (PPuup versus PPpup) with the middle 5′ section, showed that inclusion of the first 5′ section was also favoured for selection (*p* = 0.0442). Lastly, to definitively show which region was the most important, the PPpup and PPupp 5′ chimeras were competed head-to-head. Five independent transfections competing just the first and middle 5′ Udorn regions confirmed that the middle section was indeed paramount for preferential selection (*p* = 0.0008 (Student’s *t*-test)). In summary, the Udorn region spanning nucleotides 1776–2070 of the PB1 appears to be crucial in the co-selection of the Udorn PB1 and NA genes.

### 3.6. RNA-RNA Binding Studies Recapitulate Strong and Weak Interactions Predicted by Nine-Plasmid Competitive Transfections

**Figure 8:**
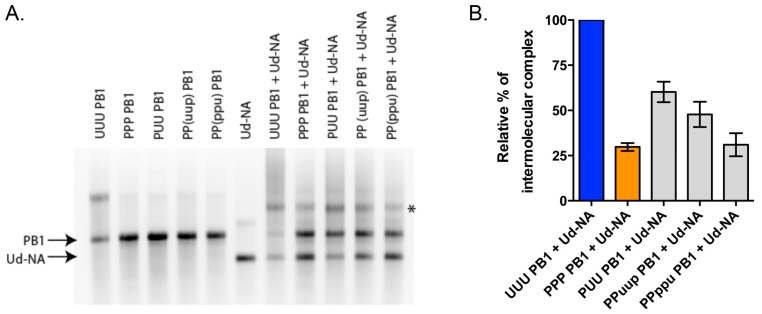
In vitro analysis of the interaction between Udorn NA vRNA and wild-type or chimeric PB1 vRNAs. (**A**) Analysis by native gel electrophoresis. Monomeric NA or PB1 vRNAs are indicated by arrows and the intermolecular complexes are marked by an asterisk; (**B**) Relative percentage of intermolecular complex. The weight fraction (%) of the RNA migrating as an intermolecular complex was calculated by dividing the weight fraction of the corresponding band by the sum of the weight fractions of all bands in the lane and normalized to the complex formed between wild-type (UUU) PB1 and Udorn NA vRNAs set to 100%. Data represent mean ± standard error (*n* = 3–4).

In order to assess whether the co-selection of the Udorn PB1 and NA genes was driven by vRNA-vRNA interactions, we used an in vitro assay that previously allowed the establishment of interaction networks between the vRNAs of the human A/Moscow/10/99 (H3N2) [17] and the avian A/Finch/England/2051/91 (H5N2) [18] influenza strains. Individually transcribed and purified vRNAs were co-incubated by pairs prior to native agarose gel electrophoresis (Figure 8A). The percentage of complex formed beween the Udorn PB1 and NA genes was our reference and was set to 100%. As expected, PR8 PB1 vRNA (PPP) interacts weakly with the Udorn NA vRNA as compared to this reference, with only 30% ± 2% of complex formed compared to the homologous PB1-NA Udorn interaction (Figure 8B). The PUU PB1 chimera, which was selected at the highest frequency (67% ± 6%) over UUU PB1 in the nine-plasmid transfection system (Figure 4A), was also the one that rescued the in vitro vRNA-vRNA interaction the best (60% ± 5%) compared to the reference. Since the last third region of PB1 displayed the strongest capacity to select the NA Udorn vRNA, we compared the ability of the PPuup and PPppu PB1 chimeras to interact, in vitro, with the Udorn NA vRNA. In agreement with results obtained from the nine-plasmid competitive transfections (Figure 7), the PPuup chimera, containing the middle section of the last 5′ region of Udorn PB1, rescued the interaction more efficiently (48% ± 6%) than the PPppu PB1 chimera (31% ± 6%), further supporting the idea that nucleotides 1776–2070 play an important role in the co-selection of the Udorn PB1 and NA genes through vRNA-vRNA interactions.

## 4. Discussion

This study was performed to locate the key region of the PB1 gene that was responsible for driving co-selection of the Udorn PB1 with the Udorn NA genes in reassortant progeny. The use of nine-plasmid competitive transfection experiments to determine preferential packaging of chimeric and wild-type PB1 RNPs was successful in narrowing down the region of potential interaction with Udorn NA RNP to approximately 1/8 the length of the complete PB1 gene. Our ability to use chimeric genes to compete with wild-type genes was facilitated by the highly conserved nature of the encoded PB1 protein that resulted in no major loss of function of the chimeric proteins nor of the replicative capacity of the viruses expressing chimeric PB1 genes. As such this approach may be applicable to other internal, highly conserved proteins of the virus. Moreover, as the genotype of progeny virions is used as the readout, the findings can be related to RNP-RNP interactions dictating the final gene constellation rather than to other vRNA-vRNA interactions that may occur during the course of replication within the infected cell.

The domain on Udorn PB1 vRNA that we have identified for potential interaction with Udorn NA vRNA, spanning nucleotides 1776–2070 of the PB1 gene, resided in the middle section of the last (5′) third of the gene, outside the identified packaging sequences [42]. Although the first 5′ section of the last third of the gene was not crucial for co-selection, it was favoured in competitions where the middle 5′ section was kept constant (PPuup versus PPpup). This implies that this first 5′ section may contribute to the co-selection of the interacting region, potentially by providing relevant nucleotide sequence across the boundary with the middle 5′ section. The boundaries were designed in nucleotide regions common to PR8 and Udorn PB1 vRNA and this particular junction had 17 nucleotides, from 1759 to 1775, in common. Hence if a non-critical part of the interacting domain resides in the first 5′ section it would conceivably be upstream of nucleotide 1759. Alternatively the first 5′ Udorn section may promote the formation of optimal secondary structures within the middle 5′ Udorn section for interaction with Udorn NA RNP. A third possibility is that this first 5′ section contains another subdominant interacting region with the Udorn NA. If there were several co-segregation signals within the gene, the strength of these signals could be hierarchical such that other interacting signals could potentially compensate for the loss of one in chimeras. This could also potentially explain why nearly all chimeric genes were preferentially selected against the wild-type PR8 gene, despite not always containing the last third of the Udorn PB1 sequence. The concept of multiple interacting regions within genes can be visualized by reference to Noda et al. [8] in supplementary movie 3 showing the 3D model of eight RNPs within a virion. Similarly, preferential selection of the Udorn PB1 gene over that of PR8 was also never 100% in competitive assays. This might indicate that regions of the PR8 PB1 vRNA can also form weak interactions with the Udorn NA or alternatively that the PR8 PB1 gene can be incorporated at a low frequency due to interactions with other genes in the supramolecular assembly complex.

We have previously shown that the eight vRNAs of both human H3N2 (A/Moscow/10/99) and avian H5N2 (A/Finch/England/2051/91) influenza A viruses form a single network of interactions in vitro [17,18]. Eight H3N2 and thirteen H5N2 interactions have been reported with only four of these in common between the two viruses. In addition to largely different networks, the sequences that were involved in the specific vRNA-vRNA interactions also differed: while vRNA-vRNA interaction sequences were mainly mapped to terminal regions in the case of the H3N2 strain [17], they essentially resided centrally for the avian H5N2 virus [18]. Similarly, RNP-RNP interactions were also visualized all along the rod-shaped structure of the RNPs in the case of the WSN viral strain [8]. Our previous report that Udorn PB1-NA co-selection is driven by interactions involving the internal coding region of the PB1 [24] supports this, and is consistent with our further refinement of the interacting region to nucleotides 1776–2070 of the PB1, which lies within this central coding region. Whether a similar co-selection of PR8 PB1 with PR8 NA exists to that of the Udorn segments is difficult to ascertain from competitive transfection experiments as the PR8 virus (containing the PR8 PB1) has a significant replicative advantage over its PR8(Ud-PB1) counterpart [24].

Experiments quantifying the ability of a subset of purified Udorn-PR8 chimeric PB1 vRNAs to form intermolecular complexes with Udorn NA vRNA were consistent with the importance of the first and middle sections of the 5′ region of the PB1 gene. This suggests that, in some instances at least, the RNA-RNA interactions measured in vitro may well predict RNP-RNP interactions within the supramolecular complex formed prior to or during budding. PB1-NA vRNA interactions were previously shown for both the A/Moscow/10/99 (H3N2) [9] and A/Finch/England/2051/91 (H5N2) [18] influenza strains, but these were relatively weak, with 15% ± 2% and 9% ± 1% of the purified RNAs forming intermolecular complexes respectively. This has made it difficult in the past to establish the importance of this interaction. In the present study, however, 52% ± 3% of the purified Udorn PB1 and NA RNA formed intermolecular complexes (*n* = 5, range 47%–65%), indicative of a strong association.

Within the 1776–2070 nucleotide domain of the PB1 we identified a number of regions where the PB1 vRNA sequence was highly complementary to that of the NA vRNA sequence, potentially allowing PB1-NA interactions. These regions were between seven and 22 nucleotides in length with complementarity between the two genes ranging from 85.7% up to 100%. At this stage, we cannot conclude whether these regions do indeed interact in RNPs, and there are too many possibilities to test them systematically using site directed mutagenesis and competition of mutated and wild-type genes, or by in vitro binding assays. Additional criteria will have to be used in order to prioritize these possible interactions, and will include those regions showing conservation of pairing within pandemic H2N2 and H3N2 isolates.

The presence of highly conserved regions of pairing in the N2 NA and the PB1 genes would explain the gene constellations arising in past pandemic viruses during their creation by reassortment. In the precursors of the 2009 H1N1 pandemic virus, the NA and PB1 genes initially co-segregated with the HA gene from the human H3N2 donor in the formation of the swine H3N2 strain. This occurred again in the formation of the swine H3N2 ‘triple reassortant’ strain, and eventually only the human H3N2 PB1 and NA genes co-segregated to form the swine H1N2 ‘triple reassortant’ strain [43]. Similarly, the PB1, NA and HA genes from the avian H2N2 donor co-segregated, when reassorting with the human H1N1 virus, to produce the 1957 H2N2 ‘Asian flu’ pandemic strain [44,45]. The PB1 of the H1N1 virus circulating in 1957 may, like that of PR8, be devoid of the correct sequence and structural characteristics to co-segregate with the N2 NA, thus favouring the incorporation of the PB1 from the avian donor. In creation of the 1968 H3N2 pandemic virus, although only the PB1 and HA genes were introduced from the avian virus donor into the existing human strain, this is not incompatible with the notion of PB1-NA co-segregation as the incoming PB1 gene from the avian donor may, as in the H2N2 pandemic virus, have had the capacity to pair with the human N2 NA.

Understanding the interactions that are involved in gene co-segregation is essential to fully appreciate the factors that govern the gene constellations of viral progeny likely to result from reassortment of viruses from human and non-human species. Those favouring the incorporation of particular polymerase genes are especially important as these genes can control the speed of replication as well as contain virulence factors that may contribute to pathogenicity. For example, some strains encode a full-length inflammatory PB1-F2 protein in the second reading frame of the PB1 gene that is associated with enhanced disease severity [46,47]. This study elucidates a mechanism of viral gene co-selection in emerging influenza viruses with pandemic potential that may govern their virulence.

## Figures and Tables

**Table 1:** Nucleotide homologies and amino acid differences between Udorn PB1 and PR8 PB1.

Region of PB1	Nucleotides	Length (nt)	Nucleotide Homology	Amino Acid Differences
Overall	1–2341	2341	83.7%	25
First third	1–716	716	85.2%	8
Middle third	717–1463	747	82.6%	9
Last third	1464–2341	878	83.7%	8

**Table 2:** Nucleotide homologies and amino acid differences between Udorn PB1 and PR8 PB1 in the 5′ third.

Region within the Last (5′) Third of PB1	Nucleotides	Length (nt)	Nucleotide Homology	Amino Acid Differences
Last 5′ third overall	1464–2341	878	83.7%	8
First third of 5′	1464–1775	312	83.9%	2
Middle third of 5′	1776–2070	295	79.5%	4
Last third of 5′	2071–2341	271	86.6%	2

## References

[1] Hsu M.T., Parvin J.D., Gupta S., Krystal M., Palese P. (1987). Genomic RNAs of influenza viruses are held in a circular conformation in virions and in infected cells by a terminal panhandle. Proc. Natl. Acad. Sci. USA.

[2] Compans R.W., Content J., Duesberg P.H. (1972). Structure of the ribonucleoprotein of influenza virus. J. Virol..

[3] Pons M.W., Schulze I.T., Hirst G.K., Hauser R. (1969). Isolation and characterization of the ribonucleoprotein of influenza virus. Virology.

[4] Pflug A., Guilligay D., Reich S., Cusack S. (2014). Structure of influenza A polymerase bound to the viral RNA promoter. Nature.

[5] Eisfeld A.J., Neumann G., Kawaoka Y. (2015). At the centre: Influenza A virus ribonucleoproteins. Nat. Rev. Microbiol..

[6] Harris A., Cardone G., Winkler D.C., Heymann J.B., Brecher M., White J.M., Steven A.C. (2006). Influenza virus pleiomorphy characterized by cryoelectron tomography. Proc. Natl. Acad. Sci. USA.

[7] Noda T., Sagara H., Yen A., Takada A., Kida H., Cheng R.H., Kawaoka Y. (2006). Architecture of ribonucleoprotein complexes in influenza A virus particles. Nature.

[8] Noda T., Sugita Y., Aoyama K., Hirase A., Kawakami E., Miyazawa A., Sagara H., Kawaoka Y. (2012). Three-dimensional analysis of ribonucleoprotein complexes in influenza A virus. Nat. Commun..

[9] Fournier E., Moules V., Essere B., Paillart J.C., Sirbat J.D., Isel C., Cavalier A., Rolland J.P., Thomas D., Lina B., Marquet R. (2012). A supramolecular assembly formed by influenza A virus genomic RNA segments. Nucleic Acids Res..

[10] Chou Y.Y., Vafabakhsh R., Doganay S., Gao Q., Ha T., Palese P. (2012). One influenza virus particle packages eight unique viral RNAs as shown by FISH analysis. Proc. Natl. Acad. Sci. USA.

[11] Inagaki A., Goto H., Kakugawa S., Ozawa M., Kawaoka Y. (2012). Competitive incorporation of homologous gene segments of influenza A virus into virions. J. Virol..

[12] Hutchinson E.C., von Kirchbach J.C., Gog J.R., Digard P. (2010). Genome packaging in influenza A virus. J. Gen. Virol..

[13] Gerber M., Isel C., Moules V., Marquet R. (2014). Selective packaging of the influenza A genome and consequences for genetic reassortment. Trends Microbiol..

[14] Marsh G.A., Hatami R., Palese P. (2007). Specific residues of the influenza A virus hemagglutinin viral RNA are important for efficient packaging into budding virions. J. Virol..

[15] Marsh G.A., Rabadan R., Levine A.J., Palese P. (2008). Highly conserved regions of influenza A virus polymerase gene segments are critical for efficient viral RNA packaging. J. Virol..

[16] Hutchinson E.C., Curran M.D., Read E.K., Gog J.R., Digard P. (2008). Mutational analysis of *cis*-acting RNA signals in segment 7 of influenza A virus. J. Virol..

[17] Fournier E., Moules V., Essere B., Paillart J.C., Sirbat J.D., Cavalier A., Rolland J.P., Thomas D., Lina B., Isel C. (2012). Interaction network linking the human H3N2 influenza A virus genomic RNA segments. Vaccine.

[18] Gavazzi C., Isel C., Fournier E., Moules V., Cavalier A., Thomas D., Lina B., Marquet R. (2013). An in vitro network of intermolecular interactions between viral RNA segments of an avian H5N2 influenza A virus: comparison with a human H3N2 virus. Nucleic Acids Res..

[19] Hatta M., Halfmann P., Wells K., Kawaoka Y. (2002). Human influenza A viral genes responsible for the restriction of its replication in duck intestine. Virology.

[20] Jackson S., Van Hoeven N., Chen L.M., Maines T.R., Cox N.J., Katz J.M., Donis R.O. (2009). Reassortment between avian H5N1 and human H3N2 influenza viruses in ferrets: A public health risk assessment. J. Virol..

[21] Li C., Hatta M., Watanabe S., Neumann G., Kawaoka Y. (2008). Compatibility among polymerase subunit proteins is a restricting factor in reassortment between equine H7N7 and human H3N2 influenza viruses. J. Virol..

[22] Essere B., Yver M., Gavazzi C., Terrier O., Isel C., Fournier E., Giroux F., Textoris J., Julien T., Socratous C. (2013). Critical role of segment-specific packaging signals in genetic reassortment of influenza A viruses. Proc. Natl. Acad. Sci. USA.

[23] Octaviani C.P., Ozawa M., Yamada S., Goto H., Kawaoka Y. (2010). High level of genetic compatibility between swine-origin H1N1 and highly pathogenic avian H5N1 influenza viruses. J. Virol..

[24] Cobbin J.C., Ong C., Verity E., Gilbertson B.P., Rockman S.P., Brown L.E. (2014). Influenza virus PB1 and neuraminidase gene segments can cosegregate during vaccine reassortment driven by interactions in the PB1 coding region. J. Virol..

[25] Chen L.M., Davis C.T., Zhou H., Cox N.J., Donis R.O. (2008). Genetic compatibility and virulence of reassortants derived from contemporary avian H5N1 and human H3N2 influenza A viruses. PLoS Pathog..

[26] Hara K., Nakazono Y., Kashiwagi T., Hamada N., Watanabe H. (2013). Co-incorporation of the PB2 and PA polymerase subunits from human H3N2 influenza virus is a critical determinant of the replication of reassortant ribonucleoprotein complexes. J. Gen. Virol..

[27] Li C., Hatta M., Nidom C.A., Muramoto Y., Watanabe S., Neumann G., Kawaoka Y. (2010). Reassortment between avian H5N1 and human H3N2 influenza viruses creates hybrid viruses with substantial virulence. Proc. Natl. Acad. Sci. USA.

[28] Octaviani C.P., Goto H., Kawaoka Y. (2011). Reassortment between seasonal H1N1 and pandemic (H1N1) 2009 influenza viruses is restricted by limited compatibility among polymerase subunits. J. Virol..

[29] Marshall N., Priyamvada L., Ende Z., Steel J., Lowen A.C. (2013). Influenza virus reassortment occurs with high frequency in the absence of segment mismatch. PLoS Pathog..

[30] Bergeron C., Valette M., Lina B., Ottmann M. (2010). Genetic content of Influenza H3N2 vaccine seeds. PLoS Curr..

[31] Fulvini A.A., Ramanunninair M., Le J., Pokorny B.A., Arroyo J.M., Silverman J., Devis R., Bucher D. (2011). Gene constellation of influenza A virus reassortants with high growth phenotype prepared as seed candidates for vaccine production. PLoS ONE.

[32] Cobbin J.C., Verity E.E., Gilbertson B.P., Rockman S.P., Brown L.E. (2013). The source of the PB1 gene in influenza vaccine reassortants selectively alters the hemagglutinin content of the resulting seed virus. J. Virol..

[33] Hoffmann E., Neumann G., Kawaoka Y., Hobom G., Webster R.G. (2000). A DNA transfection system for generation of influenza A virus from eight plasmids. Proc. Natl. Acad. Sci. USA.

[34] Tannock G.A., Paul J.A., Barry R.D. (1984). Relative immunogenicity of the cold-adapted influenza virus A/Ann Arbor/6/60 (A/AA/6/60-ca), recombinants of A/AA/6/60-ca, and parental strains with similar surface antigens. Infect. Immun..

[35] Hoffmann E., Stech J., Guan Y., Webster R.G., Perez D.R. (2001). Universal primer set for the full-length amplification of all influenza A viruses. Arch. Virol..

[36] Cavrois M., De Noronha C., Greene W.C. (2002). A sensitive and specific enzyme-based assay detecting HIV-1 virion fusion in primary T lymphocytes. Nat. Biotechnol..

[37] Naffakh N., Tomoiu A., Rameix-Welti M.A., van der Werf S. (2008). Host restriction of avian influenza viruses at the level of the ribonucleoproteins. Annu. Rev. Microbiol..

[38] Nath S.T., Nayak D.P. (1990). Function of two discrete regions is required for nuclear localization of polymerase basic protein 1 of A/WSN/33 influenza virus (H1N1). Mol. Cell. Biol..

[39] Biswas S.K., Nayak D.P. (1994). Mutational analysis of the conserved motifs of influenza A virus polymerase basic protein 1. J. Virol..

[40] Poch O., Sauvaget I., Delarue M., Tordo N. (1989). Identification of four conserved motifs among the RNA-dependent polymerase encoding elements. EMBO J..

[41] Gonzalez S., Ortin J. (1999). Distinct regions of influenza virus PB1 polymerase subunit recognize vRNA and cRNA templates. EMBO J..

[42] Fujii K., Fujii Y., Noda T., Muramoto Y., Watanabe T., Takada A., Goto H., Horimoto T., Kawaoka Y. (2005). Importance of both the coding and the segment-specific noncoding regions of the influenza A virus NS segment for its efficient incorporation into virions. J. Virol..

[43] Kingsford C., Nagarajan N., Salzberg S.L. (2009). 2009 Swine-origin influenza A (H1N1) resembles previous influenza isolates. PLoS ONE.

[44] Kawaoka Y., Krauss S., Webster R.G. (1989). Avian-to-human transmission of the PB1 gene of influenza A viruses in the 1957 and 1968 pandemics. J. Virol..

[45] Scholtissek C., Rohde W., Von Hoyningen V., Rott R. (1978). On the origin of the human influenza virus subtypes H2N2 and H3N2. Virology.

[46] Chen W., Calvo P.A., Malide D., Gibbs J., Schubert U., Bacik I., Basta S., O‘Neill R., Schickli J., Palese P. (2001). A novel influenza A virus mitochondrial protein that induces cell death. Nat. Med..

[47] McAuley J.L., Tate M.D., MacKenzie-Kludas C.J., Pinar A., Zeng W., Stutz A., Latz E., Brown L.E., Mansell A. (2013). Activation of the NLRP3 inflammasome by IAV virulence protein PB1-F2 contributes to severe pathophysiology and disease. PLoS Pathog..

